# London Hospitals "the Pay System"

**Published:** 1888-06-30

**Authors:** 


### The Hospital

A Weekly Institutional Journal of

Science, Medicine, Nursing, and Philanthropy.

*For Hospitals, Asylums, Medical Practitioners, Students, Nurses, Families, Parishes, Congregations, and the Charitable Public.*

Vol. IV. No. 92.] [June 30, 1888.

### London Hospitals and "The Pay System."

One of the most instructive and interesting discussions ever held upon any hospital question was brought to a conclusion at St. Thomas's Hospital on the 20th inst. It arose out of the able and exhaustive paper of Mr. W. Burdett-Coutts, M.P., the concluding portion of which appeared in last week's issue of The Hospital. Well sustained, representative, convincing, and admirable in tone and feeling, this discussion presented but one defect, and even this may prove in the end of great advantage to the voluntary hospitals. Although the most representative and influential of the hospital managers, honorary medical officers of general and special hospitals, hospital governors and committeemen were present and spoke, Mr. W. Burdett-Coutts was the only metropolitan Member of Parliament who attended either of the meetings. There is no excuse for the absentees; a special invitation and a copy of the paper was addressed to each metropolitan Member, and with singular unanimity they ignored both, presumably because they hold that the efficient maintenance of the hospital system in London is a matter with which they have no concern. This is a striking example of the ignorance of the feelings and interests of the people which Members of Parliament sometimes exhibit. It remains to be seen if the people of London will love to have it so, or whether those who have shown so little regard for the sick and suffering amongst their constituents will be called strictly to account for their lamentable disregard of one of the most pressing of metropolitan questions. Those who support and manage the metropolitan hospitals are an exceeding great army, and if they determine to act together, as they seem inclined to do, no metropolitan Member whom they find it necessary to oppose will retain his seat.

The urgency of this question will be apparent when it is stated that about one million and a quarter of the population obtain relief from the London hospitals and dispensaries every year. The total money value, as represented by the actual expenditure on the relief thus afforded, was £651,802, or about ten shillings and fivepence per head. Towards this expenditure an income of £560,000 was received from charitable and proprietory revenue, patients' payments, and the Hospital Sunday and Saturday Funds, leaving a deficiency of nearly £100,000. This state of affairs has been going on for some years past, and were it not that legacies produce some £200,000 on an average every three years, thus reducing the deficiency to £33,000 a-year, the whole voluntary system must shortly collapse, to the lasting disgrace of this generation, and to the eternal loss of ourselves and our descendants. This £33,000 is made good by the sale of invested funds, and the disastrous effect of this dire necessity is well seen in the case of King's College Hospital, which has been reduced to such financial distress as to imperil its longer continuance as one of the best-known of London hospitals. It is true, as we showed last week, that an extra £40,000 a-year might be obtained through the Hospital Saturday Fund, and that without much difficulty if the managers would consent to follow the wise counsels of those who have achieved such notable success in the provinces. In this way the deficiency would be liquidated altogether. Unfortunately there are still two thousand five hundred beds at present unoccupied in the London hospitals for want of an additional revenue of £100,000 a-year (*i.e.*, £40 per bed), which is required to defray the cost of making them available for the many sufferers who ought to occupy them. These beds are chiefly located in the general hospitals, which receive little or nothing from patients' payments at present, whilst the special hospitals and dispensaries obtained £40,000 from their patients during the year 1887. This fact has fixed the attention of Mr. Burdett-Coutts, and he urges with great force that the whole of these two thousand five hundred beds might be occupied to-morrow if those managers who are in a position to do so would adopt the pay system.

What, then, is the so-called pay system? It is not a system which can be properly introduced into a large endowed hospital established for the sick and deserving poor so long as the available income is large enough to defray the cost of maintaining all the beds, provided this class of patients apply in sufficient numbers to keep them constantly occupied. It is not applicable to a large voluntary hospital situated in a poor and crowded neighbourhood, where the deserving and urgent cases far exceed in number the accommodation at the disposal of the managers of the institution. The London Hospital is the most notable example of this class. Again, the pay system does not consist in demanding a registration fee from every applicant, or from the majority of applicants, who seek relief at the hospital doors, as some people appear to think. The registration fee presses everywhere unduly upon the poor, who are often unable to spare sixpence or even threepence for such a purpose. Of this system the late Sampson Gamgee, F.R.S. Edin., well and forcibly wrote:—

So long as persons know that they will be received as patients at a hospital on paying a shilling, and stating that their earnings are below a certain standard, it requires no stretch of the imagination to understand that a premium is offered to improvidence and fraud. By such a system the hospital is made a vast competitor against provident sick clubs, and the self-respect of the working population is undermined by inducements to untruthfulness, with practical immunity from detection. Under such circumstances the hospital becomes a training school not only for pauperism, but for duplicity.

Three cases may be quoted which occurred on one morning at one hospital under the registration system. A poor widow, fifty-six years of age, previously operated upon for cancer, came for treatment. She had two children, one earning 5*s.* 6*d.* per week, which, with her own earnings as charwoman, made the utmost weekly sum available for the maintenance of the family 13*s.* 6*d.* per week, but owing to her own illness, the lad's 5*s.* 6*d.* a-week was all that was left. Yet she had to pay 1*s.* at the hospital before being admitted to see the surgeon. In the second case, a lad of eighteen, earning 19*s.* per week, applied at the hospital for the treatment of a foul disease, and was registered on payment of a shilling. In the third case, a man similarly affected, earning 26*s.* a week, and having a wife only to maintain, was also accepted on payment of the shilling. Where is the charity of treating the poor half-starved widow like these two rascals, by taking a shilling, or threepence, from each as the condition precedent to admission to hospital relief? How many poor widows are kept away for want of a shilling? How many vicious and improvident men hasten to pay it, as a very cheap method indeed of getting rid of the penalties attaching to their misdeeds? No, the pay-system is not the registration fee.

What, then, is it? It is a system which reduces the number of patients to reasonable proportions, does justice to the medical staff and to the great body of the medical profession, and at the same time satisfies and relieves the applicants for relief. In England we have always so far tried, with the exception of one or two special hospitals—the Central Ear and Throat Hospital is one especially worthy of a visit for this reason—to put the cart before the horse, when (1) introducing the system of payment, or (2), endeavouring to lessen the abuses of the out and in patient departments of our hospitals. In America and the special hospitals referred to, this initial mistake has been avoided. How? By refusing to attempt to prove whether each patient is or is not a proper object of charity, and insisting instead that every applicant, whether an in or out patient, shall pay something, unless he or she can prove inability to do so, and is therefore entitled to treatment in a free bed. If our hospital managers would unanimously consent to put this system into practice to-morrow all hospital abuse would cease. Every poor person would speedily and surely obtain the hospital aid needed, and every vacant bed in the London hospitals might be occupied throughout every day in each year if the requirements of the people necessitated it. The opinions here stated are the result of long and patient inquiry, and a precise knowledge of all the facts. The meeting at St. Thomas's Hospital did well then to request the Council of the Hospitals' Association to take steps to arrange a conference of the leading hospital managers upon the pay system. Anybody who listened to Sir Sydney Waterlow, Mr. Nixon, Dr. Steele, Mr. Walsh, Mr. Burdett, and others at that meeting, and who heard Mr. Burdett-Coutts's convincing reply upon the whole case, had no doubt as to the wisdom of such a course, and we shall hope to hear of its speedily being adopted in the best interests of of all our hospitals as well as of those who resort to them for medical treatment.

